# The Influence of the Cooling Bores on Crystal Orientation and Lattice Parameter in Single-Crystalline Cored Turbine Blades

**DOI:** 10.3390/ma14143842

**Published:** 2021-07-09

**Authors:** Jacek Krawczyk, Włodzimierz Bogdanowicz, Jan Sieniawski

**Affiliations:** 1 Institute of Materials Engineering, University of Silesia in Katowice, 1a 75 Pułku Piechoty St., 41-500 Chorzów, Poland; wlodzimierz.bogdanowicz@us.edu.pl; 2 Department of Materials Science, Rzeszow University of Technology, 2 W. Pola St., 35-959 Rzeszów, Poland; jansien@prz.edu.pl

**Keywords:** single-crystalline cored blades, low-angle boundaries, lattice parameter, x-ray topography, elements segregation

## Abstract

The areas located near the cooling bores of single-crystalline cored turbine blades made of nickel-based CMSX-4 superalloy were studied. The blades were solidified by the vertical Bridgman technique in the industrial ALD furnace. Longitudinal sections of the blades were studied by Scanning Electron Microscopy, X-ray diffraction topography, X-ray diffraction measurements of the γ′-phase lattice parameter *a*, and the α angle of the primary crystal orientation. The local changes in α were analyzed in relation to the changes of the dendrite’s growth direction near the cooling bores. It was found that in the area approximately 3 ÷ 4 mm wide around the cooling bores, changes of α and *a*, both in the blade root and in the airfoil occurred. The local temperature distribution near the cooling bores formed a curved macroscopic solidification front, which caused changes in the chemical composition and, consequently, changes in the *a* value in a range of 0.002 Å to 0.014 Å. The mechanism of alloying elements segregation by tips of the dendrites on the bent solidification front was proposed. The multi-scale analysis that allows determining a relation between processes proceed both on a millimeter-scale and a micrometric and nanometric scale, was applied in the studies.

## 1. Introduction

The nickel-based superalloys belong to the class of materials specifically developed for high-temperature applications, i.e., for producing single-crystalline (SX) blades applied in high-pressure turbines for jet engines or electricity generators. Due to the harsh operating conditions, the SX blades made of superalloys must have the ability to keep high strength, creep resistance, good hot corrosion, and oxidation resistance during long-term service. Currently, one of the most widely-used nickel-based superalloys is CMSX-4. The CMSX-4 belongs to the 2nd generation of SX superalloys, consist of cubic γ-phase in the form of the net of thin channels creating the matrix, located between γ′ cubic crystals playing a reinforcement role [1,2,3,4]. The size of γ′ crystals is about 1 μm or less.

To reduce the temperature of the blades during operation, they must be cooled. Therefore, the blades are often produced with cooling bores that are formed by the use of ceramic cores in the casting mold [5,6,7,8]. Single-crystalline cored turbine blades are produced in vacuum furnaces by directional dendritic crystallization using the Bridgman technique. Both the γ and γ′ cubic phases in the casts obtained during that type of crystallization have almost the same crystal orientation. The lattice parameters of the phases in as-cast CMSX-4 superalloy are similar, for example, as presented in [9]; for the γ-phase, it is 0.3589 nm and for the γ′-phase, it is 0.3587 nm. As a result, the interfacial boundary in hypothetically-perfect superalloys is considered coherent [1,10]. Local changes in the lattice parameter can cause changes in the structure of the γ/γ′ boundary and, in consequence, of changes in its stability during the high-temperature creep of the blade [11,12].

In the vicinity of the cooling bores, the conditions of crystallization of CMSX-4, containing many alloying elements, may be different than in the rest part of the blade. Therefore, the lattice parameters of γ and γ′ solid-solutions may also be different in the areas of size about the diffusion length of alloying elements in the molten phase, which is about millimeters [13]. It may be related to the fact that the value of the γ′-phase lattice parameter in as-cast CMSX-4 superalloy can vary in a relatively wide range from 0.3587 nm [9] to 0.3579 nm [14,15]. Therefore, it is essential to examine the distribution of the lattice parameter of γ and γ′ in macroscopic areas of a few millimeters’ width around the blade bores. In this case, the use of microscopic methods, such as HRTEM or HRSEM, is hard or impossible because they require small samples and allow the analysis of microscopic areas. However, it can be made possible by macroscopic X-ray diffraction methods using diffractometers in which the area of the sample coverage by the incident beam is about 1 mm in diameter, and the measurements may proceed in single-pass with sufficiently high accuracy, enabling the analysis of changes in the lattice parameter. These conditions are met by the Ω-scan method, for which both the α angle and lattice parameter measurements can be performed during a single-pass measurement over the entire studied area with sufficiently good accuracy [16]. On the other hand, a separate determination of a lattice parameter of γ and γ′ phases using X-ray macroscopic methods is difficult due to their very similar value. For CMSX-4 SX casts, the volume fraction of the γ′-phase is about 70% [1]. Therefore, the influence of the γ-phase on the position of reflexes in a diffraction pattern is low and although both phases are included in the diffraction area covered by the incident X-ray beam, the position of the reflexes allows one to determine the γ′-phase lattice parameter and its spatial distribution in the cast areas of millimeters in size.

**Figure 1:**
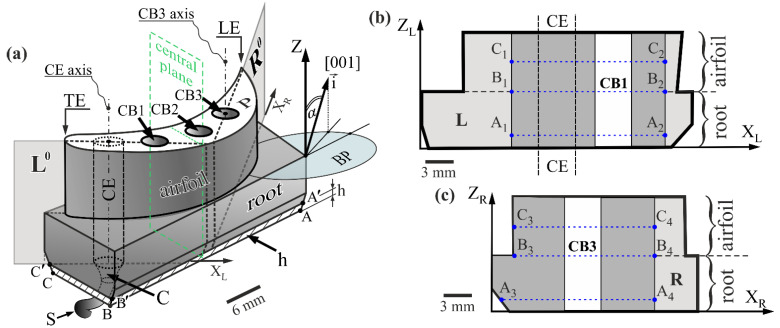
The scheme of the blade with the location of the cutting planes L^0^ and R^0^ and scheme of the graphical definition of primary crystal orientation angular component α (**a**) and shapes of the surfaces L (**b**) and R (**c**) of the sample. Z—blade axis, perpendicular to surface ABC of the root, CB1, CB2, CB3—cooling bores, S—selector, C—continuer, CE—continuer extension, h—5-mm thick bottom root layer of the unsteady dendrite growth, LE and TE—leading and trailing edge of airfoil, P—top airfoil surface, perpendicular to the Z-axis, BP—base plane parallel to ABC surface of the root and perpendicular to Z, $\vec{i}$—unit vector. The α angle is enlarged for figure clarity.

The production technology based on the Bridgman technique allows for obtaining blades that have the required crystallographic orientation of [001]-type [1] almost parallel to the Z-axis of the blade (Figure 1) with a low value of the α angle of a primary crystal orientation. The proper selection of technological parameters, such as the temperature gradient and crystallization rate, will allow for obtaining the blades with high structural perfection and good strength and mechanical properties [17,18,19,20]. It was stated that any deviation of [001] direction from the Z-axis defined by the α angle (Figure 1) causes deterioration in blade quality [1,21,22]. Therefore, the value of the α angle and its spatial distribution in the blade significantly influence the mechanical and strength properties of blades. In cored turbine blades, the temperature gradient and the content of alloying elements near the bores’ side surface may be different than in the remaining part of the blade. This can significantly change the direction of the dendrites’ growth and, consequently, the value of the α angle. Therefore, it is extremely important to examine the α angle distribution in areas located near the cooling bores.

Producing the single-crystalline blades, particularly cored blades made of nickel-based superalloys, is complicated, mainly due to the complex shape of blade surfaces, both external and internal, and the specificity of dendrite growth during directional crystallization by the Bridgman method [1]. For this reason, various growth defects (i.e., formed during the growth of dendrites) can be created during crystallization near the cooling bores. Some of them, such as low-angle boundaries (LABs), are not eliminated by further processing [23]. A too-large number of growth defects may reduce the strength and creep resistance of the blade [24,25,26,27,28]. Therefore, in this work, it was decided to use the X-ray diffraction topography method, which can visualize both LABs, dendrite bends, and changes in their crystal orientation, as well as local changes in the lattice parameter of the γ′ solid-solution. Using this method to compare the orientation of adjacent sample areas over the entire tested surface and the α angle and lattice parameter measurements by the Ω-scan method seems very promising. This type of research has not been conducted so far.

The mold cores, which form the bores, may influence heat dissipation during the Bridgman process and, consequently, the growth of the dendrites during directional crystallization of the blades. For this reason, it is essential to analyze the distribution of the α angle of primary orientation and lattice parameter of the γ′-phase of a blade in an as-cast state.

There are no data in the literature on the measurement of the lattice parameter and the misorientation during the single-pass measurement on the sample area of tens of millimeters, as well as the comparison of the results with the data obtained using the X-ray topography method, which is an application novelty. These analyses can be used to develop the production technology of cooled single-crystalline blades to extend their service life by improving the strength properties dependent on crystal misorientation and heterogeneity of the chemical composition.

Considering the above, the study was aimed to analyze the as-cast structure of SX cored turbine blades in the vicinity of the cooling bores concerning changes of the crystal orientation described by the α angle and changes in the γ′-phase lattice parameter depending on the distance from the bores. This goal has been achieved by a comprehensive analysis of the structural heterogeneity of the blades, both on the macroscopic scale concerning fragments of a dendritic array consisting of several or several dozen dendrites (scale of the order of millimeters), on the microscopic scale concerning single dendrites (scale of hundreds of micrometers), and on the scale of the unit cell of the γ′-phase (tenths of a nanometer). Such a multi-scale analysis seems very useful for the analysis of single-crystalline multicomponent dendritic superalloys.

## 2. Materials and Methods

The single-crystalline cored turbine blades made of a CMSX-4 nickel-based superalloy were studied in an as-cast state. The nominal chemical composition in wt.% was as follows: 5.6 Al, 1.0 Ti, 6.5 Ta, 6.5 Cr, 0.6 Mo, 6.0 W, 9.0 Co, 3.0 Re, 0.1 Hf, less 0.002 C, and Ni bal. The model blades containing three cooling bores CB1, CB2, and CB3 (Figure 1a), were obtained by directional solidification at a withdrawal rate of 3 mm/min. using the Bridgman technique. The standard axial temperature gradient of the furnace growth chamber was G_0_ = 16 K/cm [29]. An asymmetrically-positioned spiral selector, S, with the continuer, C, was used to give the [001] crystal orientation of the cast (Figure 1a). The cylindrical area bounded by the projection of the continuer perimeter into the blade was named the continuer extension (CE) area. The industrial ALD Vacuum Technologies furnace was applied to crystallize the blades, which is the equipment of the Research and Development Laboratory for Aerospace Materials, Rzeszów University of Technology, Poland.

The sample for the tests was prepared from each of the five obtained blades in several steps. In the first step, the bottom root layer h of a thickness of about 5 mm (Figure 1a), in which dendritic crystallization could occur under unsteady conditions [30], was cut off and excluded from the study. In the second step, the sample for tests was prepared by cutting off the fragment of the blade along the planes L^0^ and R^0^ (Figure 1), which were parallel to the Z-axis of the blade (Figure 1). The L^0^ plane was arranged to pass through the trailing edge (TE) of the airfoil and CE axis, intersecting the CB1 (Figure 1a). The R^0^ plane was set to pass through the leading edge (LE) of the airfoil and CB3 axis (Figure 1a). Each obtained sample has lateral surfaces L and R of a shape presented in Figure 1b,c, respectively. The metallographic sections of studied surfaces were prepared using the standard procedure for superalloys [31]. Only fragments of the L and R surfaces located around CB1 and CB2 were analyzed. These fragments are marked in dark gray in Figure 1b,c. The considered fragment of the L surface, located to the left of the CB1, was extended with the CE area. It was assumed that there is the least concentration of defects in the CE, so that it could be the reference area to those located near the CB1. The surfaces of CB1 and CB3 were excluded from the measurements due to previous scanning of the tested regions by the laser system and selecting the measurement points only on the surface from which it is possible to obtain X-ray diffraction. The laser scanning procedure is implemented in the applied diffractometer.

The X-ray diffraction topograms were recorded from the studied fragments of the L and R surfaces (dark grey areas in Figure 1b,c). The topograms were recorded by narrowing down the incident beam to the analyzed fragments. This allowed the background of the topograms to be lowered. Determination of the primary crystal orientation was performed by measurements of the α angle (Figure 1a) defining the deviation of the [001] direction from the *Z*-axis of the blade. The measurements of the α angle and lattice parameter of γ′-phase hereinafter referred to as *a*, were performed at points forming lines perpendicular to CB1 and CB2 surfaces, by the Ω-scan method using dedicated EFG Freiberg Instruments X-ray diffractometer [16]. The standard error of the *a* measurement was 5 × 10^−4^ Å, and the α angle can be evaluated with a mean error of 0.006°. The measurement lines were denoted as A_1_-A_2_, B_1_-B_2_, and C_1_-C_2_ for fragments of the L surface, as well as A_3_-A_4_, B_3_-B_4_, and C_3_-C_4_ for fragments of the R surface. Lines A_1_-A_2_ and A_3_-A_4_ were located in the lower part of the root, where the dendrites’ steady growth began [30]. Lines B_1_-B_2_ and B_3_-B_4_ were positioned at the level of the connection between the root and airfoil. Lines C_1_-C_2_ and C_3_-C_4_ were located in the center of the airfoil height (Figure 1b,c). The step between measurement points on the lines was 0.5 mm. Characteristic CuKα radiation was used. The collimated incident beam covered an area of 0.8 mm in diameter on the tested surface. It was assumed that the unit vector $\vec{i}$ (Figure 1a) arranged parallel to the [001] direction defines the growth direction of the primary dendrite arms [1,14,30].

Using the X-ray diffraction topography, the crystal misorientation of local areas in studied fragments of the L and R surfaces was visualized. Before recording the topograms, the L and R surfaces were crystallographically oriented by the Laue diffraction using the X-ray diffractometer of the RIGAKU/EFG XRT-100CCM system provided by EFG Freiberg Instruments (Freiberg, Germany). The divergent X-ray beam of characteristic CuKα radiation was applied for the X-ray topography study using the PANalytical Microfocus diffractometer (Alamelo, The Nederlands). The anode of the microfocus source has a focal spot dimension of 20 × 20 μm^2^. The topograms were recorded in the AGFA Structurix X-ray film. The sample coupled with the X-ray film oscillated around the Bragg angle within ±4°about the Z-axis. The topograms were recorded with the use of 113-type reflections. The method is described in detail in [32,33,34].

## 3. Results and Discussion

**Figure 2:**
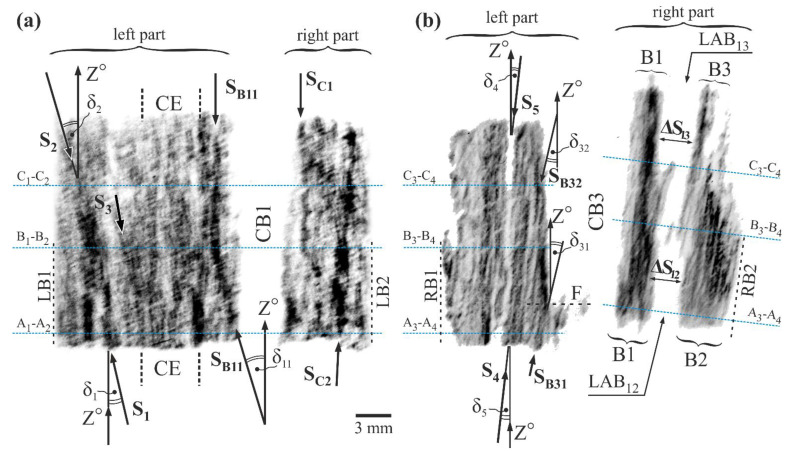
X-ray diffraction topograms, obtained from fragments of the surfaces L (**a**) and R (**b**). CB1 and CB3—cooling bores; CuKα radiation, 113-type reflection; Z°-axes are parallel to the blade’s Z-axis (Figure 1a). Blue dashed lines indicate images of the α and *a_γ′_* measurement line traces.

Figure 2 shows two exemplary topograms recorded from the analyzed fragments of the surfaces L and R. The left part of the topogram presented in Figure 2a was recorded from the left fragment of the surface L located on the left side of CB1, while the right part of the topogram was recorded from the right fragment of the surface L located on the right side of CB1. The topogram consisting of left and right parts, recorded from left and right fragments of the surface R, is shown in Figure 2b. Alternating bright and dark contrast bands mostly tilted to the left to Z°-type axes parallel to the blade’s Z-axis are visible in the left part of the topogram presented in Figure 2a. The dark bands marked by S_1_, S_2_, S_3_, and S_B11_ arrows can be used as an example. In the right part of the topogram presented in Figure 2a, the contrast bands are not tilted but are parallel to Z°, for example, bands S_C1_ and S_C2_. This part of the topogram was recorded from the right fragment of surface L located near the central plane of the sample that divides the blade root into two equal parts (Figure 1a).

The contrast bands similar to that presented in the topogram of the left fragment of the L surface, but mainly finer and tilted to the right to the Z°-axes, are visible in the left and right part of the topogram recorded from the left and right fragment of the surface R (Figure 2b). For example, dark fine band S_4_ and the bright extensive wider band S_5_, and extensive bands LAB_12_ and LAB_13_, as well as fine dark bands S_B31_ and S_B32_. The dark contrast bands visualize single dendrites’ primary arms or groups because the heavy alloying elements with higher atomic scattering factors (like Re, W, and Mo) segregate into dendrite arms. The tilt of the dendrites images in the topograms, to the left in Figure 2a and to the right in Figure 2b, as well as the parallel orientation of the dendrites images to the Z-axis of the blade in the right part of the topogram from Figure 2a corresponding to the central plane of the blade, are consistent with the so-called “fanning effect” described in [21,35]. The arrangement of dark contrast bands, placed quite far to the left of CB1, for example, bands marked by the arrows S_1_, S_2_, and S_3_ (Figure 2a), indicates that the dendrites located at a higher distance from the CB1 more often grew inclined to the Z°-axes that are parallel to the Z-axis of the blade. The inclination of bands S_1_ and S_2_ may be described by the angles δ_1_ and δ_2_ (Figure 2a). The curved dark contrast bands are visible near the edge of the topograms parts corresponding to the left sidewall of the cooling bore CB1 (S_B11_—Figure 2a) and cooling bore CB3 (S_B31_, S_B32_—Figure 2b). The contrast bands S_B11_, S_B31_, and S_B32_ are curved so that they change the direction from inclined to parallel to the Z°-axes (and also Z-axis). For example, the band S_B11_, which is inclined by the angle δ_11_ in the area below the B_1_-B_2_ line, is parallel to the Z°-axis above the line. A similar change in the direction of the bands is observed near CB3. An example may be the S_B31_ band, which, near line A_3_-A_4_, is inclined at the δ_31_ angle and above line F, is parallel to Z°. Another example is the S_B32_ band, which below line C_3_-C_4_ is inclined at δ_32_ and above that line, is parallel to Z°. Thus, it can be concluded that in the vicinity of the cooling bores, the force-directing of the dendrites occurs parallel to the surface of the bores, i.e., parallel to the Z-axis of the blade.

The topogram, obtained from the right fragment of the surface R located on the right hand of the CB3, has a specific distribution of the finer contrast bands that are grouped into the B1, B2, and B3 groups that are mutually shifted. The examples of the shift are marked in Figure 2b as ΔS_12_ and ΔS_13_. Each group represents a single subgrain separated by low-angle boundaries (LABs) [33]. The LAB_13_ can be distinguished between the subgrains B1 and B3 and the LAB_12_—between the subgrains B1 and B2. In the right part of the topogram presented in Figure 2b, the blur contrast near the CB3, LAB_12_, and LAB_13_ is visible. It can be related to stresses created near the subgrain boundaries and near the sidewall of CB3.

In the left part of Figure 2b and left and right parts of Figure 2a, images of borders of CB3, CB1, and images of borders of topograms (LB1, LB2, and RB1) are vertical and parallel to the direction of crystallization Z°. This means that the part of the sample represented by the L surface (Figure 2a) and the left part of the R surface (Figure 2b) consists of low-misoriented dendrites. On the other hand, the image inclination of the right part of the R surface (Figure 2b) suggests a significant misorientation of the sample part located to the right of CB3 in relation to the remaining parts of the sample.

Topograms contain information on both the δ angles of inclination of dendritic bands or individual dendrites from the vertical direction of crystallization Z°, and information on the angles of mutual misorientation of adjacent areas of the sample (e.g., subgrains or individual dendrites). In the case of a low misorientation of sample areas, the dendrite bends defined by the δ angle can be visible in the topogram. This occurs for the sample part represented by its entire L surface and the part of the sample represented by the left fragment of the R surface (Figure 2a and left fragment of Figure 2b). The δ angle defining the dendrite growth direction is higher, the higher the α angle measured by the EFG diffractometer, but not equal to the α angle. In Figure 2b, the inclination of the right border of CB3 and right border of the topogram (RB2) in relation to the left border of CB3 and Z°, represents a significant misorientation of the entire part of the sample situated to the right of CB3 in relation to those parts of it that lie to the left of CB3. It was found that the misorientation value is high for this type of blade, which was presented in [33].

On the right part of the topogram presented in Figure 2b, images of traces of the measurement lines C3-C4, B3-B4, and A3-A4 are marked as perpendicular to the right border of CB3 and right border of the topogram (RB2) because de facto, the measurements were made along the line that was perpendicular to CB3 (see Figure 1).

**Figure 3:**
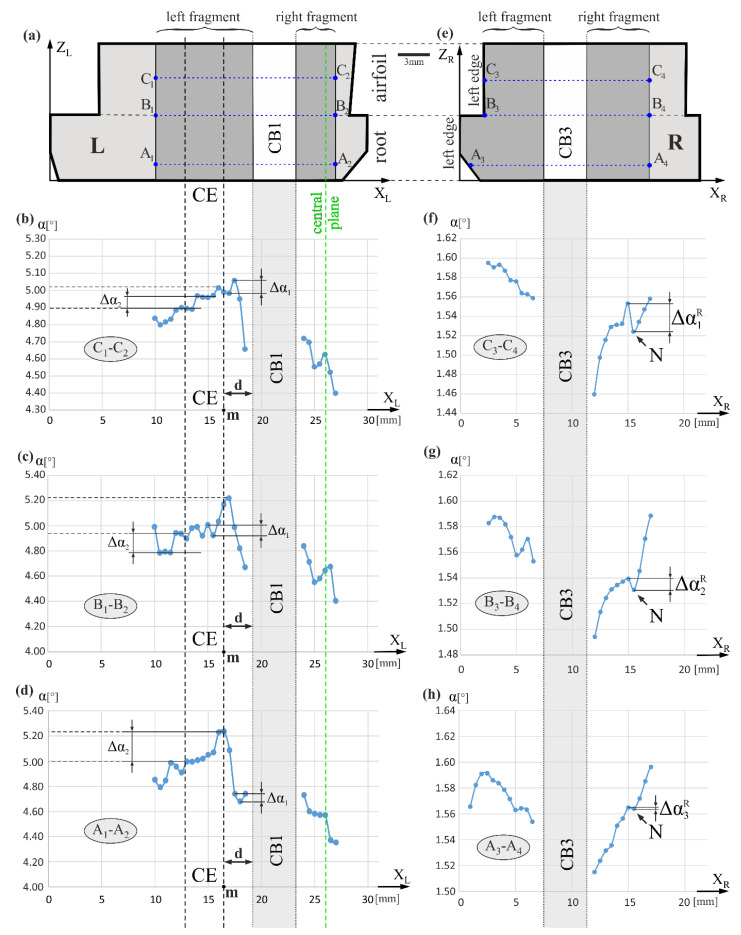
Scheme of the arrangement of the α angle measurement lines (**a**,**e**) and the α angle values distribution in the fragments L (**a**–**d**) and R (**e**–**h**) surfaces along these lines perpendicular to the axes X_L_, X_R_, and blade axis Z, as well as, perpendicular to the sidewall of CB1 and CB3 cooling bores in their vicinity. For simplicity, the labels of the measurement axes are replaced by parallel axes X_L_ and X_R_.

Figure 3 shows the arrangement of the measurement lines and the exemplary distributions of the α angle value along lines A_1_-A_2_, B_1_-B_2_, and C_1_-C_2_, and A_3_-A_4_, B_3_-B_4_, and C_3_-C_4_. All the lines are perpendicular to the axes Z_L_, Z_R_, and Z of the blade (Figure 1b,c), as well as parallel to the X_L_ and X_R_ axes, therefore, the labels of the measurement lines axes are also marked as X_L_ and X_R_ for simplicity. Corresponding measurement lines are drawn in the topograms presented in Figure 2. The lines A_3_-A_4_, B_3_-B_4_, and C_3_-C_4_ on the right side of CB3 are inclined due to the inclination of the topogram part, because the sample surface—from which the part of the topogram was recorded—was strongly misoriented. In Figure 3b–d, it is visible that inside the CE area located in the left fragment of the surface L, along all lines, the α value changes in the range of about 4.9° ÷ 5.2°. On the right side of the CE or on its right border with coordinate *m*, the α values increase slightly. Inside area *d*, 3 mm in width, near the CB1, the α decreases to the value of about 4.65° for all diagrams in Figure 3b–d. Generally, the areas of the α angle changes (increase and decrease) and have a width of about 4 mm. The diagrams show that the α(X_L_) relationship have a Δα_1_ fluctuation with approximate values of 0.1° for some neighboring point and Δα_2_ step changes with values ranging from 0.07 to 0.2° for adjacent point groups forming short plateaus. In the first case, it is probably related to the changes in the orientation of individual neighboring dendrites. In the second case, it is related to the changes in the orientation of adjacent groups of dendrites. To the right of the CB1 sidewall, a decrease of the α value visible in the area *d* is continued with some disturbances. For the fragments of surface R (Figure 3f–h), in the area on the left of the CB3 (left fragment of R, Figure 3e), the α value generally decreases near the CB3, but in the area on the right side (right fragment of R), it increases with some disturbances in the point N of a maximal value of about 0.027° ($\Delta \alpha_{1}^{R}$ in Figure 2f). The disturbances are probably related to the differences in the crystal orientation of neighboring subgrains B1 and B2 or B1 and B3 (Figure 2b). The α changes that may correspond to the LAB_13_ and LAB_12_ boundaries are much smaller than those that can be determined from the topograms based on the shift ΔS_12_ or ΔS_13_. The latter can be estimated at 1.7° ÷ 2.3° using the method presented in Reference [32]. These differences result from the fact that the ΔS_12_ or ΔS_13_ shifts are related to the rotation of the dendrites about an axis almost parallel to Z° or Z_R_, while the α change is related to the inclination of the dendrites relative to this axis. Primary arms of the dendrites, growing toward Z° (Z_R_) may rotate around Z° about an angle of several degrees, and their inclination to the axis Z° (Z_R_) may not change at all.

**Figure 4:**
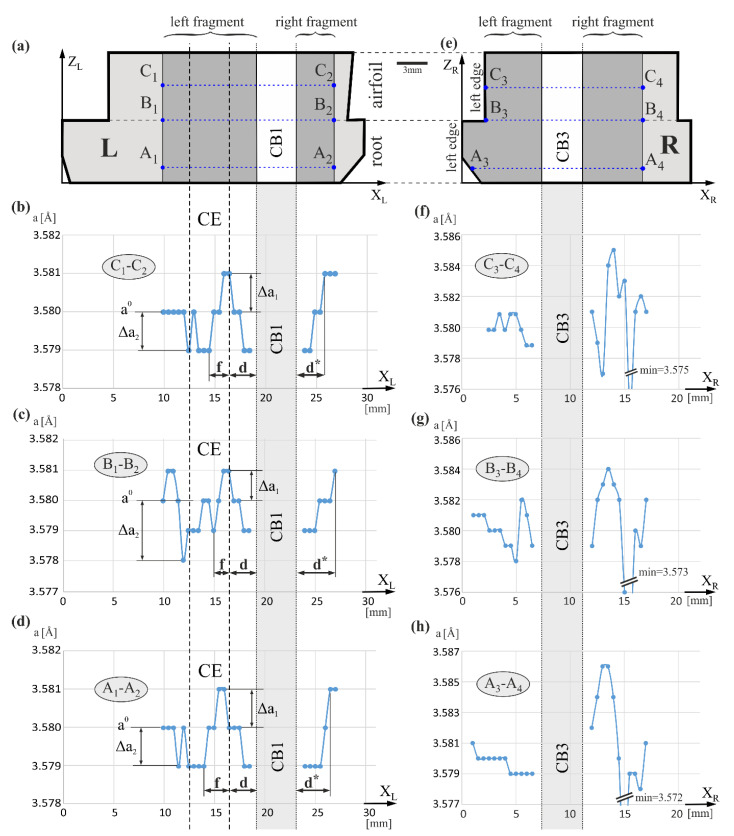
Scheme of the arrangement of the lattice parameter measurement lines (**a**,**e**) and lattice parameter *a* values distribution in fragments of the L (**a**–**d**) and R (**e**–**h**) surfaces along selected lines perpendicular to the axes X_L_, X_R_, and the blade axis Z, as well as, perpendicular to the sidewall of CB1 and CB3 cooling bores in their vicinity. For simplicity, the labels of the measurement axes are replaced by parallel axes X_L_ and X_R_.

Figure 4 presents the arrangement of the measurement lines and exemplary value distributions of the lattice parameter *a* along these lines. The axes of the measurement lines A_1_-A_2_, B_1_-B_2_, and C_1_-C_2_ (Figure 4a) and A_3_-A_4_, B_3_-B_4_, and C_3_-C_4_ (Figure 4e) are marked with the same labels as the X_L_ and X_R_ axes due to their parallelism and for simplification. Characteristic changes of the *a* value are visible near the sidewall of cooling bore CB1 in the areas *d* and d* of about 3 mm in width (Figure 4b–d). A decrease of *a* on the left side and an increase on the right side of the CB1 sidewall was observed. The range of the *a* changes is comparable on both sides of the cooling bore. This means that near the cooling bore CB1, there is a reduction in the *a* value. On the left of area *d*, in area *f* of a width of about 1 mm, which is part of the CE, *a* decreases with decreasing the X_L_. In the left fragment of CE there are quite significant Δ*a* fluctuations, but generally, the *a* value is smaller compared to the right fragment of CE. In some distance to the left of the left CE border, fluctuations generally decline and the value *a* is stabilized at the *a_0_*, as shown in Figure 4b,d. In the case of the function shown in Figure 4c, recorded on line B_1_-B_2_, the trend of *a* stabilization is poorly visible, which may be related to the existence of a low-angle boundary crossed by line B_1_-B_2_ and/or significant casting stresses near the CE border [23]. The changes of Δ*a_1_* and Δ*a_2_* type, visible in Figure 4b–d, may be related to changes in the chemical composition of neighboring individual dendrites or/and their groups, which in turn may be related to the dendritic segregation of alloying elements that occurs during crystallization. Groups of points in graphs with a practically constant *a* value may correspond to the set of parallel dendrites forming wider contrast bands on topograms (for example, S_1_, S_2_—Figure 2a). Generally, the *a_γ′_* value of the γ′-phase varies in a range of 0.002 ÷ 0.014 Å for all measurement fragments of the L and R surfaces.

The mold core, forming CB1, was connected with the bottom part of the mold. That caused additional heat transfer along with that core, the reduction of the temperature near the core, and local bends of the macroscopic crystallization front in the vicinity of the core, also around the bores of the blade. The bends of the crystallization front may cause macroscopic segregation of the alloying components and local variations of the *a* parameter in the areas *d*, d*, and *f*. The mechanism of such a segregation will be described hereinafter. Changes of the *a* value in fragments of the R surface, especially on the right side of CB3 (Figure 4f–h), are of a different, more complex character. The presence of LAB_12_ and LAB_13_ bands or a bright, fairly wide S5 band visible in the topogram presented in Figure 2b, visualizing the low-angle boundaries with high misorientation angles, which causes large changes of the lattice parameter against the background of a smaller change of *a* induced by bends of the crystallization front near CB3, similar to that near CB1. The overlapping of these two effects creates complex changes in the *a*(X_R_) relationship.

Growth of dendrites in the required [001] crystallographic direction is often changed by the walls of the mold, resulting in bending of dendrites in areas near the blade surface [36]. A somewhat similar effect may be observed near the inner walls of the mold, e.g., near the sidewall of CB1 and CB3. The bending of dendrites is clearly visible in the X-ray topograms (Figure 2) because X-ray topography is sensitive to even minimal angular changes. In the topograms, changes in the direction of the bands of increased contrast near CB1 and CB3 correspond to changes in the growth direction of the dendrites. In the case of CB1, the S_B11_ band—initially inclined at δ_11_ to the Z° axis above line A_1_-A_2_—in a later stage of crystallization, approximately at line B_1_-B_2_, curves and becomes parallel to the Z° axis. A similar effect is observed on the left side of CB3 for the S_B31_ band with the initial inclination angle of δ_31_ and for the S_B32_ band with the initial inclination angle of δ_32_. The first of these bands is parallel to the Z°-axis above level F (Figure 2b) and the second one—above line C_3_-C_4_. The tendency of the dendrite orientation along Z°-axes near the CB3 sidewall is additionally described by decreasing the α values in the α(X_R_) graphs on the left side of CB3 (Figure 3f–h). These graphs also show a reduction in the α value when distance to CB3 decreases from the right side. The situation is different for the dendrites to the right of CB1. Since the α value increases when the distance to the right of the CB1 sidewall decreases, contrary to the above-described changes, it can be related to the additionally “fanning effect” [21].

**Figure 5:**
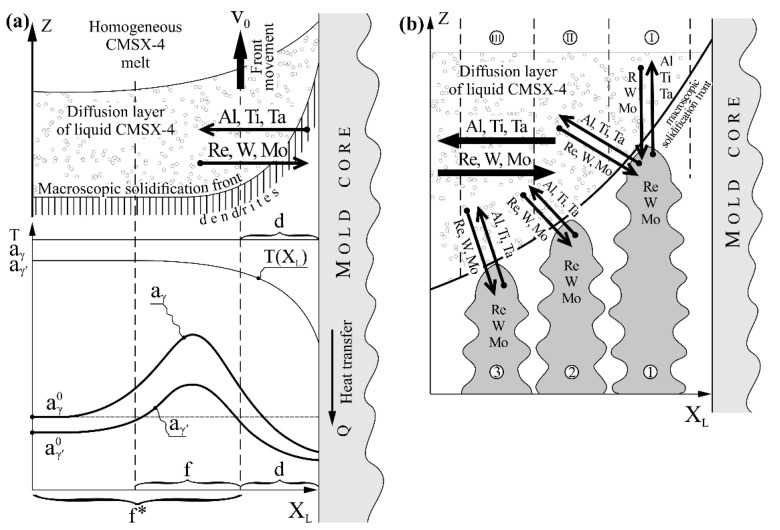
Scheme of formation of alloying elements distribution parallel to the X_L_ axis near the mold core for curved macroscopic crystallization front, as well as, distribution of lattice parameters *a_γ_* and *a_γ′_* (**a**) and scheme of the Al, Ti, and Ta and Re, W, and Mo diffusion fluxes formation by dendrite tips (**b**).

Figure 5a shows the hypothetical distribution of the lattice parameter of the solidifying γ_I_-phase and γ′-phase created by γ_I_ -> γ_II_ + γ′ transformation in the solid, along the X_L_ axis. The γ_I_ is a γ-phase with the chemical composition corresponding to the mushy zone with the coexistence of liquid and γ_I_-phase in the Ni-Al phase equilibrium diagram, and γ_II_ is the γ-phase with the chemical composition corresponding to the zone of coexistence of γ′ and γ phases in this diagram [1]. In addition, a formation scheme of the alloying elements flux in the diffusion layer of the liquid CMSX-4 ahead of the curved crystallization front, moving at the rate v_0_ towards the Z-axis, is presented. Figure 5b shows the mechanism for “transferring” the Al, Ti, and Ta in liquid by a curved front from the mold core side, and Re, W, and Mo in the opposite direction (Figure 5a). It is known that Al, Ti, and Ta segregate during crystallization into interdendritic areas and Re, Mo, and W—to the dendrites [1]. The tip of dendrite 1 (Figure 5b) solidifies higher than dendrite 2. As a result, Al, Ti, and Ta are transferred in front of the dendrite tip from layer I of the liquid into layer II. This recapturing process is repeated by the tip of the dendrite 2, then by dendrite 3, and by many following dendrite tips. As a result of the process, the content of Al, Ti, and Ta in the liquid area of *d*-width (Figure 5a) decreases, and in the area of width *f*, it increases. At the same time, Re, W, and Mo are captured from the diffusion area of the liquid into the tips of dendrites. This capture is more intense when the crystallization front is strongly curved because it occurs from a wide range of directions of the diffusion layer—from the front of the dendrite tip and from its left side, exposed by the curved front. That process illustrates vertical and inclined arrows, presented in Figure 5b for dendrite 1. The capturing of Re, W, and Mo, and recapturing of Al, Ti, and Ta by the dendrite tips is the reason for creating lateral diffusion fluxes. The fluxes, illustrated by the horizontal arrows, are the reason for a decrease in Al, Ti, and Ta and the increase in Re, W, and Mo in the layer of *d*-width (Figure 5a). A change in the chemical composition in a liquid layer of *d*-width causes a change in the chemical composition of the γ-phase that crystallizes in a blade’s d-layer (inside the dendrite and interdendritic areas). According to Vegard’s law, a decrease in Al, Ti, and Ta content and increase Re, W, and Mo content causes the change in the lattice parameter of this γ-phase [1], described by Equation (1):

(1) $$a_{\gamma }=3.524+0.110\;X_{Cr}^{\gamma }+0.478\;X_{Mo}^{\gamma }+0.444\;X_{W}^{\gamma }+0.441X_{Re}^{\gamma }+0.179\;X_{Al}^{\gamma }+0.422\;X_{Ti}^{\gamma }+0.700\;X_{Ta}^{\gamma }\;\left[Å\right]$$

where [$X_{i}^{\gamma }]=\frac{Å}{\mathrm{at}.\;\%}$.

From Equation (1), it can be concluded that the Al, Ti, and Ta flux from a liquid *d*-layer decreases *a_γ_*, and the Re, W, and Mo flux inside the *d*-layer increases *a_γ_*. However, it can be shown that the contribution of the Al, Ti, and Ta flux in the change of *a_γ_* is more essential, and therefore *a_γ_* in the *d*-layer will be decreased. Such a conclusion can be drawn based on the below-mentioned analysis.

Table 1 presents the mass and atomic content of alloying elements for the CMSX-4 superalloy and the percentage difference in atomic diameter from Ni in the γ-phase, as well as the Vegard’s coefficients of alloying elements for γ-phase. The data were collected using [1,37,38].

It may be concluded from Table 1 that Cr has the lowest Vegard’s coefficient with a relative difference in atomic diameter D_X_-D_Ni_/D_Ni_ of 3%. Co has a smaller parameter at 1%, thus it should have an even lower Vegard’s coefficient. For this reason, Cr and Co can be omitted, and then Equation (1) can take the form:

(2) $$a_{\gamma }=3.524+0.179X_{Al}^{\gamma }+0.422X_{Ti}^{\gamma }+0.700X_{Ta}^{\gamma }+0.441X_{Re}^{\gamma }+0.444X_{W}^{\gamma }+0.478X_{Mo}^{\gamma }\;[Å].$$

Compare the influence of atoms of two fluxes, Al, Ti, and Ta, and Re, W, and Mo, on the *a_γ_* parameter. Equation (2) and Table 1 show that Ta has almost twice the Vegard’s coefficient compared to W and that the contents of both elements in CMSX-4 are comparable (2.17 at.% and 2.11 at.%). Vegard’s coefficients for Ti and Mo are similar, and the Ti content in liquid CMSX-4 is almost three times higher (1.28 at.% and 0.38 at.%). On the other hand, Al has a Vegard’s coefficient that is about two times smaller than Re, but its atomic content is about 12 times higher (12.58 at.% and 0.94 at.%). In addition, the rate of Al diffusion is higher. It follows that the segregation, described by an increase in the atomic concentration of Al, Ti, and Ta in area *f* and its reduction in area *d*, will have a much stronger effect on the lattice parameter of γ-phase crystallizing in those areas than the inverse segregation of Re, W, and Mo. Therefore, in the area *d*, the lattice parameter of the γ-phase (*a_γ_*) is smaller, and in area *f*, it is larger compared to the areas where there is no curved crystallization front and where the value of the lattice parameter is $a_{\gamma }^{0}$ (Figure 5a). However, in the presented study, the lattice parameter of the γ′-phase was measured.

The differences in the lattice parameter of the γ′-phase in areas *d* and *f* should be similar to that of the γ-phase. In other words, a type of segregation of the alloying elements in the γ-phase described above, also in a similar way affects the chemical composition and parameter *a_γ′_* of the γ′-phase. Additionally, it is known that as a result of the γ_I_ -> γ_II_ + γ′ transformation, Re, W, and Mo, the concentration of which is high in area *d*, diffuse from phase γ′ to γ_II_—according to the partition coefficient presented in [9]. This diffusion process further reduces the parameter *a_γ′_* inside the area *d* of the blade, according to Vegard’s law [1]:

(3) aγ′=3.570−0.004XCrγ′+0.208XMoγ′+0.194XWγ′+0.262XReγ′+0.258XTiγ′+0.500XTaγ′ [Å].

According to the partition coefficient presented in [9], Ti and Ta diffuse to the γ′-phase due to phase transformation. The process weakly influences the increase of *a_γ′_* in area *d* because the content of these elements is lower. In area *f*, where the concentration of Al, Ti, and Ta is increased (Figure 5a) due to the phase transformation, these elements segregate into the γ′-phase—according to the partition coefficient—which increases the lattice parameter *a_γ′_* in area *f*. On the other hand, the diffusion of Re, W, and Mo from the γ′-phase has a weak effect on the reduction of *a_γ′_* in area *f*, because the content of these elements in area *f* is small. Since the γ′-phase lattice parameter was measured during the experiment, its reduction in area *d* and increase in area *f* were noticeable.

The location of the selector in the blade cast may have a significant influence on the growth of the dendrites and on the changes of the α angle and lattice parameter of the γ′-phase around the cooling bores. In the studied blades, the selector with the continuer was located (in the section view) between the TE side of the airfoil and cooling bore CB1 (Figure 1). The continuer extension area (CE) is devoid of essential distortions of the dendritic array that appear outside it, as indicated by a more homogenous contrast on the topograms inside CE. However, the α angle in CE is the highest (Figure 3), which means the highest inclination of the dendrites growing to the left, and so the inclination of the [001]-type direction in the blade. Due to the asymmetric location of the selector, the single grain that survived in the selector is not oriented strictly in the direction of 001. The “fanning effect” causes the inclination of the dendrites, and so the [001]-type direction, to the left. Considering the “fanning effect”, the selector should be placed centrally on the blade’s cross-section.

The highest number of local misorientation defects, such as LABs, appears on the right side of the surface R, farthest from the CE. There are several sub-grains with higher misorientation angles related to a significant mutual rotation of adjacent sub-grains about an axis approximately parallel to the Z-axis. Moreover, the α angle value in the right side of surface R is lower than in the CE area (Figure 3). It is also related to the “fanning effect” that causes a reduction of the α value by inclination dendrites to the right and by compensation of inclination to the left given by dendrites from the CE area. However, increasing the number of local misorientation defects of the dendritic array is observed with increasing the distance to the CE area.

The growth of the primary dendrites from the selector continuer takes place not parallel to the Z-axis but inclined to the left. The cooling bores limit the dendrites’ sloping growth, causing local directing of the primary dendrite arms to the Z-axis direction by bending them near the walls of the bores. The effect is related to increased vertical heat transfer through the mold’s cores and local increase in a vertical temperature gradient.

The asymmetrical selector location on the cross-section of blades causes non-axial orientation of the dendrites to the Z-axis of the blade according to the “fanning effect”. Paradoxically, the cores that are joined to the lower surface of the mold locally “correct” non-axially arrangement of the dendrites located in their vicinity, but at the same time create local inhomogeneity of the α angle and the lattice parameter of the γ′-phase, which is related to the heterogeneity of the chemical composition. Such inhomogeneity may cause a concentration of stress in local areas of blades during the growth of the dendrites.

## 4. Conclusions

The spatial distribution of the α angle of primary crystal orientation and lattice parameter *a* of γ′-phase in cored turbine blades crystallized with an asymmetrically located selector is not homogenous. The local changes of *a*, both in the root and airfoil, occur near the side surface of the cooling bores in areas with a width of 3 ÷ 4 mm. It may be related to the segregation of the alloying elements caused by tips of the dendrites-forming local bends of the macroscopic crystallization front near the cooling bores. Local changes of the α angle, both in the root and airfoil, occur in areas with a width of 3 ÷ 4 mm near the sidewall of cooling bores. The dendrites that grow tilted to the sidewall of the cooling bores change the growth direction to parallel to them.

The multi-scale analysis of the structure, both on the macroscopic scale concerning fragments of a dendritic array consisting of several or several dozen dendrites (scale of the order of millimeters), on the microscopic scale concerning single dendrites (scale of hundreds of micrometers), and on the scale of the unit cell of the γ′-phase (tenths of a nanometer) is very useful for analyzing the relationship between the structural defects of single-crystalline, dendritic, and multicomponent superalloys.

Additional changes in the α angle, related to the “fanning effect” in the whole blade, overlap with the above-described local changes of the α. It was found that both in the root and airfoil of the blades, low-angle boundaries (LABs) with a higher misorientation angle of 1.7° ÷ 2.3° occur at a greater distance from the selector extension area (SE). In addition, the local changes of the lattice parameter *a* are higher in areas located away from the SE. These are unfavorable effects caused by the asymmetric location of the selector in the blade casts.

The influence of the mold cores on the growth of the dendrites and formation of the structural inhomogeneity in the blades probably may be reduced by applying the mold cores made in the form of ceramic tubes with less thermal conductivity. This will reduce the heat flux dissipated directionally through the cores.

## Figures and Tables

**Table 1:** Mass and atomic content of alloying elements for the CMSX-4 superalloy and percentage difference in atomic diameter from Ni, as well as the Vegard’s coefficients of alloying elements for the γ-phase.

	Al	Ti	Ta	Re	W	Mo	Cr	Co	Ni
mass%	5.6	1.0	6.5	3.0	6.4	0.6	6.5	9.5	balanced
at.%	12.58	1.29	2.17	0.94	2.11	0.38	7.45	9.86	62.17
D_X_-D_Ni_/D_Ni_ [%]	+6	+9	+18	+10	+13	+12	+3	+1	0
Vegard coeff. for γ [Å/at.%]	0.179	0.422	0.700	0.441	0.444	0.478	0.11		
